# Factors Related to Psychological Distress during the First Stage of the COVID-19 Pandemic on the Chilean Population

**DOI:** 10.3390/jcm10215137

**Published:** 2021-10-31

**Authors:** Carlos Ruiz-Frutos, Diemen Delgado-García, Mónica Ortega-Moreno, Daniel Duclos-Bastías, Dánica Escobar-Gómez, Juan Jesús García-Iglesias, Juan Gómez-Salgado

**Affiliations:** 1 Department of Sociology, Social Work and Public Health, Faculty of Labour Sciences, University of Huelva, 21007 Huelva, Spain; frutos@uhu.es (C.R.-F.); juanjesus.garcia@dstso.uhu.es (J.J.G.-I.); 2 Safety and Health Postgraduate Programme, Universidad Espíritu Santo, Guayaquil 092301, Ecuador; 3 IMPACTCOVID-19 Chile Research Project, Universidad de Aconcagua, Los Andes 2102660, Chile; diemen.delgado@uac.cl; 4 Department of Economy, University of Huelva, 21007 Huelva, Spain; 5 School of Physical Education, Faculty of Philosophy and Education, Pontificia Universidad Católica de Valparaíso, Valparaíso 2374631, Chile; daniel.duclos@pucv.cl (D.D.-B.); danica.escobar@pucv.cl (D.E.-G.); 6 Faculty of Education Sciences, University of Cádiz, 11519 Cádiz, Spain

**Keywords:** COVID-19 emergency, psychological distress, stress disorder, preventive measures, mental health, Chile

## Abstract

The health effects of COVID-19 continue to raise doubts today. In some areas, such as mental health, these doubts have scarcely been addressed. The present study analyses the effects on psychological distress during the first phase of the pandemic in Chile. A cross-sectional descriptive study was performed by using a questionnaire validated in Spain and adapted for Chile. Between 22 April and 16 December 2020, 3227 questionnaires were collected from the 16 regions of Chile, using non-probabilistic snowball sampling. Bivariate analysis and binary logistic regression were performed. The variables that could predict psychological distress during the COVID-19 pandemic in Chile were: having a poor self-perception of health OR = 4.038, 95% CI = (2.831, 5.758); being younger than 29 OR = 2.287, 95% CI = (1.893, 2.762); having diarrhea OR = 2.093, 95% CI = (1.414, 3.098); having headache OR = 2.019, 95% CI = (1.662, 2.453); being a woman OR = 1.638, 95% CI = (1.363, 1.967); having muscle pain OR = 1.439, 95% CI = (1.114, 1.859); and having had casual contact with an infected person OR = 1.410, 95% CI = (1.138, 1.747). In Chile, with a better social, economic, cultural, and health environment compared to neighboring countries, there has been a high percentage of psychological distress. It is time to prioritize measures to safeguard the mental health of Chileans, especially focused on the most vulnerable population according to our results, i.e., young women with poorer health status.

## 1. Introduction

COVID-19, with an onset at the end of 2019 in Wuhan, China, was declared by the WHO as an international public health emergency in January 2020 [1] and as a global pandemic in March 2020 [2]. It spread rapidly throughout Latin American countries, leading the WHO to declare the region as an epicenter of the pandemic in May 2020 [3].

It has been estimated that in Latin American countries, including Chile, despite the fact that preventive measures against COVID-19 were implemented without delay, these have not had the expected effects due to, among other causes, deficiencies in the contact tracking and follow-up system, as well as problems prior to the pandemic, such as the characteristics of the health system, social inequalities, high rates of informal employment, and little or late establishment of economic support measures [4].

Regarding health inequities, proven to exist in Chile, and the finding of higher mortality rates in the metropolitan area of Chile, observing a direct association between mortality from COVID-19 and poverty [5]. The social determinants of health, in particular the multidimensional poverty index and the use of public transport, play an important role in explaining the differences in outcomes [6], both in the incidence of COVID-19 and in mortality [7].

On the other hand, the influence of the economy on health is an aspect of special relevance. In this regard, analyses of the effects of the containment measures on Chile’s economic activity have been carried out [8]. One of these analyses, that covered the response to the pandemic by several Latin American governments, including Chile, has found that while wealthier municipalities introduce technological innovations comparable to those in developed countries, smaller or less advantaged areas have more difficulty maintaining service delivery while in an unprecedented socio-economic context, as is the one experienced during the pandemic [9]. However, it has been found that this health situation has not affected all population sectors in Chile equally [10]. Thus, as for the indigenous population, the vulnerability indicators previously detected have increased since it is a group that already had inequalities in health [11,12].

Overall, the impact of the COVID-19 pandemic in Chile has been significant. According to official data, as of 19 May 2021, 1,292,096 cases had been confirmed (6.81% of the general population), with 27,934 deaths and 39.78% of the country’s total population fully vaccinated. This last figure is much higher than in the countries that surround Chile as well as some of the European continent, such as Spain [13].

Regarding the effects of the physiological symptoms of the disease, cough, dyspnea, anosmia, generalized fatigue, and respiratory type problems predominate, as well as an increased risk of thromboembolic events as a result of the inflammatory state generated by the cytokine storm [14,15,16,17], although many infected persons remain asymptomatic [18]. As for the adoption of preventive measures, it has been shown that they have contributed to reducing the impact of the pandemic in those countries where they have been adopted early, with Chile having carried out a high number of diagnostic tests [19]. Several studies have been carried out to determine the factors that influence the use of preventive measures to prevent COVID-19 and their association with the development of psychological distress. It has been determined that “hand washing” was the most widely used preventive measure in Spain during the first phase of the pandemic [20], that preventive measures will depend on the risk perception acquired [21], and that psychological distress depends on the high perceived costs of adherence to the preventive measures [22], uncomfortable feeling of wearing personal protective equipment, or the public ignorance of preventive measures [23].

One of the main characteristics of the COVID-19 pandemic is that it has been classified as a “psychological pandemic”, with great effects on the mental health of the general population and, especially, on health professionals, who have been directly involved in the care of patients with COVID-19 [16,24,25,26]. In this sense, greater psychological affectation has been shown in professionals who were quarantined, who worked in COVID-19 units, or had a family member or friend infected with COVID-19. These effects manifested more through greater depression, anxiety, frustration, fear, and post-traumatic stress than in those persons who did not have such experience [27].

Regarding the general population, women [24], young people, the self-employed, individuals with previous psychological issues whose follow-up was interrupted [28], immigrants, or workers of essential activities and in contact with the public were the most affected strata [26,29]. Higher levels of stress, depression, or anxiety have been found in these groups [15,24,30,31,32], as well as somatization [24] and psychiatric disorders [17], especially in those patients with previous mental problems [33,34]. However, a study conducted in 21 countries, including Chile, did not find an increase in the number of suicides in the first months of the pandemic [35]. The psychological impact of the pandemic has manifested itself even in countries with low infection rates and good initial management of the outbreak, such as South Korea. In this population, symptoms of stress, anxiety, depression, and sleeping difficulties have been reported, albeit to a minimal or moderate degree. [36].

Among these findings, it is noteworthy that, although older people have a higher risk of suffering from serious illness due to COVID-19, they show fewer negative effects on their emotional health than young people [37], something also proven in other countries [38]. However, the elderly population is more vulnerable to stigma related to COVID-19. As an at-risk population, they are known to be more likely to be affected by the disease and this can lead to stigmatization, resulting in social rejection, isolation, and discrimination [39].

These works, carried out at the international level, provide an overview of the state of mental health among the population during the COVID-19 pandemic. However, there is currently no data that records this situation in Chile, that is, the psychological impact of the pandemic on the Chilean population has not been described. Thus, the novelty of this work lies in being the first to study this problem in Chile. The results would help measure and describe the impact of the pandemic, guide strategies for managing and addressing the crisis, and design interventions adapted to the needs of the Chilean population, as well as to develop a prevention plan for similar future situations.

Therefore, the objective of this article is to present the effects of the first wave of the COVID-19 pandemic on the mental health of Chileans, in particular, in the development of psychological distress. In this sense, it is intended to analyze the possible association with sociodemographic variables, perception of health, physical symptoms, having required health care, having received diagnostic tests, adoption of preventive measures, or contact history, among others.

## 2. Materials and Methods

### 2.1. Design and Sample

The design was a cross-sectional descriptive study, using a questionnaire previously validated and cross-culturally adapted to the Chilean environment. The sample was made up of the Chilean population, accessed through the non-probability sampling methodology snowballing method, the same methodology used in the study carried out in “Europe on Living, Working and COVID-19” by Eurofound [40].

In order to participate in the study, it was necessary to meet the following inclusion criteria: reside in Chile, be over 18 years old, and accept the informed consent. The estimated sample size was 3294, with 95% confidence level, a precision of 1.8% and a loss adjustment of 10%. Finally, the loss was 8.14%, leaving a sample size of 3227.

### 2.2. Materials

A questionnaire previously validated in Spain [20] was used, composed of several previously validated instruments adapted to the linguistic and cultural use of the language in Chile so that no question posed any difficulty of understanding. For this purpose, a panel of experts consisting of psychologists, epidemiologists, doctors, nurses, and public health experts was selected.

The questionnaire consists of three parts. In the first part, sociodemographic data were included: sex, level of education, age, work situation, cohabitation, having children or pets at home, having some degree of disability, and being under lockdown at home. In the second, Goldberg’s general health questionnaire (GHQ-12) [41] was used to measure the level of mental health and psychological well-being. This questionnaire consisted of 12 items and four answer options, in which 1 meant *better than usual* or *more than usual*, 2 *same as usual*, 3 *less than usual* or *less so*, and 4 *much less than usual* or *much less*, as regards the positive items. As for the negative ones, 1 meant *not at all*, 2 *no more than usual*, 3 *rather more than usual* or *rather more*, and 4 *much more than usual* or *much more*. 0 points were assigned to the first two options, and 1 point to the last two, with a total score ranging from 0 to 12. The cut-off point established for the general population was three, considering scores greater than or equal to 3 as psychological distress. In the third part, questions were related to the perception of COVID-19 symptoms and the history of contacts during the last 14 days: headache or sore throat, cough, fever, rhinitis, dizziness, myalgia, shortness of breath, chills, or diarrhea. Questions about taking medication, suffering from chronic illness, or having required medical attention or hospitalization during the last 14 days were also included. These items were assessed with a yes/no dichotomous answer. The possible history of contact during the last 14 days was measured by means of three items: possible contact for more than 15 min less than two meters away, casual contact with confirmed infected persons, and contact with persons or materials suspected of being infected; also, the existence of an infected relative diagnosed by a diagnostic test. Participants could respond categorically to these items with three possible options: yes, no, or doesn’t know.

Another variable collected was self-perception of their level of health during the last two weeks, this being a well-known indicator for predicting mortality [42]. It was measured with five levels of response, from very bad to very good, grouped for the final analysis into two categories, bad and excellent.

Finally, the preventive measures adopted were also included in the questions, using a Likert scale with five response options categorized from never to always with respect to the frequency with which they were performed: wearing a mask regardless of the presence or absence of symptoms; washing hands immediately after coughing, touching the nose, or sneezing; washing hands after touching potentially contaminated objects; washing hands with hydroalcoholic solution; washing hands with soap and water; covering the mouth with the elbow when coughing or sneezing; avoiding sharing utensils (e.g., spoon) during meals; leaving at least a meter and a half of separation from others. One point was assigned to the *never* answer, 2 points to *rarely*, 3 points to *sometimes*, 4 to *almost always*, and 5 to *always*. Thus, each item could score between 1 and 5, and the total score of the scale would range from 8 to 40.

### 2.3. Procedure

The Qualtrics^®^ storage and survey platform (Qualtrics, Provo, UT, USA) was used to collect the information through an online questionnaire. For its dissemination, the collaboration of universities and scientific societies was requested, and social networks and interviews in the press were used. The questionnaire was disseminated online and through the social media in order to reach a larger number of participants.

### 2.4. Data Analysis

Frequencies, means and/or standard deviations were presented depending on the type of variable. The relationship of the qualitative variables with the psychological distress was analyzed through the Chi-squared test, also obtaining the odds ratio (OR) with the associated confidence intervals. The association between the different scores was analyzed by the Student’s *t*-test for independent samples.

Finally, a binary logistic regression was performed that allowed for an assessment model to be built to study the presence or absence of psychological distress and identify those variables that played a relevant role. OR values indicate the strength of the relationship with psychological distress; the further away from 1, the stronger the relationship is.

To verify the appropriateness of the model, different measures of goodness of fit were used: the Hosmer–Lemeshow test, percentage of correctly classified values, sensitivity, and specificity. The inclusion of the variables was carried out with tests of statistical significance, the OR were estimated, and the confidence intervals were facilitated. The OR values indicate the strength of the relationship with psychological distress. All analyses were carried out with the SPSS 26.0 statistical software (IBM, New York, NY, USA).

### 2.5. Ethical Principles

At the beginning of the questionnaire, participants received information about the objectives of the study and were asked to provide their written informed consent prior to answering. The data were recorded anonymously, treated confidentially and met the ethical principles established in the Declaration of Helsinki (Fortaleza, 2013) and all the legal regulations in force on data protection and regulation of human research processes in Chile. The study has been authorized by the Ethics Committee of the University of Aconcagua in Chile (Santiago, UAC-22 April 2020) and in Spain by the Research Ethics Committee of Huelva, belonging to the Regional Ministry of Health of Andalusia (PI 036/20).

## 3. Results

### 3.1. Sociodemographic Data

A pilot test was carried out with 57 people, diverse regarding their profession, educational level, geographical scope, age, and sex, and where a Cronbach’s alpha coefficient of 0.910 was obtained, good psychometric properties, and no understanding problems. Questionnaires were received from the 16 Regions of Chile, with higher response rates from Valparaiso and Santiago. Records were obtained from 38 of the 43 types of classified occupations, with 21% of health professionals. A total of 3227 questionnaires were received between 21 April and 24 December 2020.

As can be seen in Table 1, there is a higher percentage of women (63.40%), people with university level education or higher studies (59.25%), young people (51% being 29 years old or less), living without a partner (70.31%), without children (63.43%), living in a house with an exterior view (house with balcony, terrace, yard, or garden) (86.6%), having a pet (71.4%), working in private companies (46.27%), being health professionals (21.2%), being in strict confinement or in confinement except for purchase-work (81.7%).

### 3.2. Psychological Distress

78.83% of the sample had psychological distress (PD), following the ≥3 cut-off point of the GHQ-12. The overall mean of the 12 items (GHQ-12) was M = 6.16 (SD = 3.76), with a reliability coefficient of the optimal measurement scale of Cronbach’s α = 0.910 (Table 2).

As shown in Table 2, the items with the highest score are: *Have you felt constantly overwhelmed and stressed?* M = 3.08 (SD = 0.91); *Have you been able to enjoy your normal daily activities?* M = 2.92 (SD = 0.89); *Have you been able to concentrate well on what you were doing?* M = 2.86 (SD = 0.80); and *Have your worries made you lose a lot of sleep?* M = 2.82 (SD = 0.99).

On the contrary, the items with a lower valuation have been: *Have you thought that you are a worthless person?* M = 1.68 (SD = 1.01); *Do you feel reasonably happy considering all the circumstances?* M = 2.37 (SD = 0.85) and *Have you felt capable of making decisions?* M = 2.29 (SD = 0.80).

### 3.3. Sociodemographic Data and Their Relationship with Psychological Distress

Table 1 shows how PD is more present among women OR = 1.916, 95% CI = (1.613, 2.273); aged 29 or younger OR = 2.651, 95% CI = (2.219, 3.168); without a couple OR = 1.959, 95% CI = (1.643, 2.336); with lower educational level (secondary school or lower) OR = 2.132, 95% CI = (1.771, 2.567); living in a house without balcony/terrace/yard/garden OR = 1.631, 95% CI = (1.232, 2.160); without children OR = 2.222, 95% CI = (1.873, 2.639); and not being a health professional OR = 1.385, 95% CI = (1.062, 1.580). Higher PD was found among public employees (78.7%) than among workers of private companies (70.6%) and self-employed workers (61.8%), *p* < 0.001.

It is not observed that having a pet or any degree of disability is associated with the development of PD. Being in strict confinement or being able to go out only for purchase or work is indeed associated with the level of PD (Table 1).

### 3.4. Physical Symptoms, Perception of Health, Health-Related Variables and Psychological Distress

76.8% claimed to have an excellent self-perceived health. A bad perception, versus an excellent health perception, is associated with a higher level of PD, with an OR = 6.803, 95% CI = (4.808, 9.524) (Table 3).

The most frequent symptoms were: headache (53.6%), rhinitis (34.8%), muscle pain (26.2%), cough (20.0%), and sore throat (19.4%). Having any of the symptoms is associated with developing PD, except for fever (1.4% had it). The symptoms with the highest percentages of high PD are: diarrhea (91.6%), dizziness (90.9%), fever (89.1%), muscle pain (89.0%), breathing difficulties (88.7%), chills (88.4%), and headache (87.5%); all results had *p* < 0.001 (Table 3).

29.3% reported having a chronic disease and 37.2% were taking medication. During the last 14 days, 8.7% had required medical care and 0.6% had required hospital care. No association was observed between these variables and developing PD.

### 3.5. Psychological Distress and Contact History

The proportion of participants who claimed they had not been in contact for more than 15 min and within 2 m away with an infected person was 78.1%. 71.9% reported not having been in casual contact with an infected person and 61.3% had not had any contact with a person or material suspected of being infected. 14.3% stated that they had undergone a diagnostic test (Table 4).

These contact histories, except for having received a diagnostic test, were associated with having developed PD, finding statistical significance (*p* < 0.05) and an OR greater than 1.3 (Table 4).

### 3.6. Psychological Distress and Preventive Measures

The preventive measure with a higher mean score was “wearing a mask regardless of the presence of symptoms” (M = 4.77; SD = 0.62), followed by “washing hands with soap and water” (M = 4.75; SD = 0.52). A statistically significant association has been found between having PD and the use of the following preventive measures: “washing hands after coughing, touching the nose, or sneezing”; “avoiding sharing utensils”, both with *p* < 0.001; “leaving at least one and a half metres away”, *p* = 0.002; “wash hands with soap and water”, *p* = 0.003; and “washing hands with soap and water”, *p* = 0.025 (Table 5).

### 3.7. Prediction of Psychological Distress during the Pandemic

The variables that can predict PD during the COVID-19 pandemic in Chile according to the binary logistic regression are: having a bad self-perception of health OR = 4.038, 95% CI = (2.831, 5.758); being younger than 29 OR = 2.287, 95% CI = (1.893, 2.762); having diarrhea OR = 2.093, 95% CI = (1.414, 3.098); having headache OR = 2.019, 95% CI = (1.662, 2.453); being a woman OR = 1.638, 95% CI = (1.363, 1.967); having muscle pain OR = 1.439, 95% CI = (1.114, 1.859), and having had casual contact with an infected person OR = 1.410, 95% CI = (1.138, 1.747).

These variables correctly predict and classify 79.5% of psychological distress, with a sensitivity/specificity of 17.4/96.2, R2 = 0.126; Hosmer–Lemoshov test χ2 = 13.514 (*p* = 0.095) and Omnibus test χ2 = 433.575 (*p* < 0.001).

## 4. Discussion

As previously mentioned, the development of psychological effects, especially PD, is an event that is closely related to the occurrence of the COVID-19 pandemic. In the present study, a high percentage of people with a high level of PD (78.83%) has been observed, with a ≥ 3 cut-off point in the GHQ-12, data that are above those obtained in Spain (71.98%) with a similar study methodology and cut-off point [20]. The choice of cut-off point at this given level (≥3) should be considered when comparing with other studies. These results are consistent with those obtained in previous similar studies [43,44,45].

It has been suggested that once key responses are adopted at the public health level, such as diagnostic testing, contact tracking, lockdown, and the management of confirmed cases of COVID-19, perhaps it is time to prioritize measures to safeguard the mental health of Chileans [46]. Even more, with the knowledge that the percentage of the population fully vaccinated has achieved higher levels than in other countries of the same geographical environment [13]. However, the speed in vaccinating the population may have caused an unjustified optimism that led to the abandonment of preventive measures after the first dose of the vaccine and, as the PAHO Director stated, “the vaccine alone is not going to stop the pandemic” [47].

Therefore, it seems that there is still time to prevent serious effects on mental health, since studies conducted in 21 countries, including Chile, have not observed, for instance, that high levels of PD, as the ones found in the study at hand, have led to an increase in the number of suicides in the first months of the pandemic [35].

In a somewhat contradictory way, a high percentage of the studied population claims to have an excellent self-perception of health during the last 14 days (76.8%), being the variable that mostly predicts PD, in the same way that it is known that PD is a highly reliable predictor of mortality [42]. This leads to a certain degree of optimism if measures are taken in time to enhance protective factors and mitigate the effects of the foreseeable economic recession resulting from this health crisis [35].

Differences have been seen between the symptoms found in a group of Latin American countries, the most common being cough (60.1%), fatigue/tiredness (52.0%), sore throat (50.3%), and fever (44.2%) [16], while in the present study, the most frequent symptoms have been: headache (53.6%), rhinitis (34.8%), muscle pain (26.2%), cough (20.0%), and sore throat (19.4%). In a study carried out in Spain with the same methodology, both headache and muscle pain had similar figures, but sore throat and cough had significantly higher values, while rhinitis occurred at higher rates in Chile [40]. This difference could be explained to some extent by the sociodemographic variables, the different information received by the populations of these countries, or even by being in different climatic seasons derived from belonging to the northern or southern hemisphere. In the study at hand, the three symptoms that predict PD are diarrhea, headache, and muscle pain.

The sex and age variables, as well as living with children, predict the level of PD, as has been also referred to in the literature [24,28,29,38,48,49,50,51].

Having a history of contact is associated with the presence of PD, both through contact with contaminated people or material, or with infected relatives, but it is the variable “having been in casual contact with an infected person” that mostly predicts PD, something already corroborated in previous studies [27].

It is well known that the proper and early use of preventive measures to avoid COVID-19 produces benefits in terms of health [52]. In this sense, the preventive measures with a higher valuation are: “wearing a mask regardless of the presence of symptoms” and “washing hands with soap and water”. This second measure coincides with the study carried out in Spain [20], but the use of a mask receives a much higher value in Chile. This could be explained by the fact that the data collection was carried out in Spain in earlier dates than in Chile, and during the first months of the pandemic, in Spain there was no such recommendation for the widespread use of the mask, and there were even supply problems.

On the other hand, in other studies carried out in Chile, it was found that males and people under 60 years of age were the most compliant groups with the preventive measures established by the Government, while in Colombia or Ecuador, it was women and the elderly who complied the most [48].

When designing public health policies, the stressors identified in the literature, which are related to financial, academic, and family concerns, should be taken into account, the stress of confinement being a clear predictor of mental health [24], and obviously influencing the conditions of the home of the confined person. In the present study, we have found that PD is associated with living in houses with no exterior exit (house without a balcony, terrace, yard, or garden), identifying this as the most potentially stressful type of housing, an issue that would be convenient to consider when planning urban development.

Living without a partner and not having children are other stressor variables identified in this study, which highlight the importance of family support in pandemic situations, as the importance of social support has been observed with other health problems [53].

Compared with all six Latin American countries that have been studied (Argentina, Brazil, Chile, Colombia, Mexico, and Peru), Chile is the country shown to have positive variables against COVID-19, since it has a lower percentage of poverty, higher level of schooling, and the best health system. On the contrary, it has a high percentage of the population over 65 years of age, and it adopted a partial and not total lockdown, unlike other surrounding countries [54]. Another positive factor is the fast pace of vaccination administration, higher not only than other Latin American countries, but even higher than some European countries, such as Spain [13].

On the other hand, health professionals, as previously mentioned, are a group with high levels of PD and other indicators of poor mental health [16,33], while in the present study, show a lower percentage of PD than non-health professionals. One possible explanation may be the invisibility of non-health workers in situations of risk (e.g., delivery staff, cleaners, drivers, law enforcement bodies), who, being essential jobs, have had to continue performing their work during lockdown, being in contact with contaminated people or objects and for whom vaccination has not been established as a priority, as has been the case with health professionals. Other explanations may be the effect of teleworking, increased lockdown, or greater effects on the economy, with its consequent impact on PD [55] or the social support they have had during the pandemic [56], although the latter has not been observed in other studies [57].

In this socio-economic context, and based on the levels of PD found in the study, the adoption of preventive measures focused on the prevention of possible mental effects in high-risk populations is considered of special relevance.

The limitations of this research are the same as those of all descriptive studies without randomized sampling, along with those related to online access to data, which leave out groups without internet access or without knowledge for its use. This can be seen in that 59.25% of the participants had a university level of education or higher. Moreover, online data collection does not guarantee a homogeneous territorial distribution, with some areas of the country being more affected than others. However, the characteristics of the study advised applying the sampling used. In addition, this research has been carried out with the same methodology in 18 countries in Latin America and Europe, which will facilitate comparisons in the near future that will allow increasing the available evidence on the issue studied. This methodology was also chosen for the Eurofound study and promoted by the European Union [40], but its results did not allow for causal associations to be obtained and will require future studies with more appropriate designs to test the hypotheses detected. Another limitation is the difficulty in answering certain questions, such as “whether having touched contaminated objects” or “having been in contact with sick people during the previous 14 days”. Similarly, the GHQ is a general measure of mental health, although it is a widely used and highly reliable indicator. In our study, it obtained an α-Cronbach’s score = 0.910.

## 5. Conclusions

We have been able to verify that in Chile, a country with theoretically high levels of protection against the COVID-19 pandemic, including a high percentage of vaccination, higher than those of neighboring countries, the percentage of people with psychological distress is very high in the population studied.

It has been possible to identify variables associated with PD such as being a woman, being under 29 years of age, and with a low level of education, vulnerable groups already described in other countries. Furthermore, the influence of family support becomes visible by observing that living without a partner or not having children act as variables associated with PD. The type of housing is another factor to consider when it comes to urban planning, and to establish the importance of having a house with exterior exit (balcony/terrace/yard) to reduce the PD generated in pandemics that force diverse degrees of lockdown of the population.

Something seemingly contradictory has been detected; non-health professionals showed a higher level of PD than health professionals, a group that is the subject of most studies regarding the effects of the COVID-19 pandemic. This can help visualize the group of workers of essential activities, who have had to continue to develop their work during the pandemic and, therefore, have also been exposed to contact with contaminated people or objects, but with a lower level of prioritization when it comes to vaccination.

For these reasons, the need to prioritize the establishment of programs that safeguard the mental health of Chileans before these negative effects evolve into irremediable situations or become difficult to address is evident. In this study, vulnerable groups with whom intervention would be efficient and effective, have been identified.

## Figures and Tables

**Table 1:** Association between sociodemographic variables and psychological distress during the COVID-19 pandemic.

	TOTAL (*N* = 3227)					
		GHQ				
	*N* (%)	YES (*N* = 2544)	NO (*N* = 683)	χ2	*p*	Odds Ratio (CI 95%)
Sex						1.916 (1.613, 2.273)
Female	2045 (63.4)	82.9	17.1	56.224	<0.001	
Male	1182 (36.6)	71.7	28.3			
Age * (*N* = 3224)						2.651 (2.219, 3.168)
29 years or younger	1654 (51.30)	86.5	13.5	119.842	<0.001	
Older than 29	1570 (48.70)	70.8	29.2			
Marital status						1.959 (1.643, 2.336)
Single	2269 (70.31)	82.4	17.6	57.282	<0.001	
With a couple	958 (29.69)	70.5	29.5			
Educational level						2.132 (1.771, 2.567)
Upper secondary school or lower	1315 (40.75)	85.9	14.1	65.563	<0.001	
University or higher	1912 (59.25)	74.0	26.0			
Type of dwelling						1.631 (1.232, 2.160)
Apartment/House without balcony/terrace/yard	431 (13.4)	85.2	14.8	11.893	0.001	
Apartment/House with balcony/terrace/yard/garden	2796 (86.6)	77.9	22.1			
You are (*N* = 1502)						-
Independent worker	233 (15.51)	61.8	38.2	25.712	<0.001	
Public employer	574 (38.22)	78.7	21.3			
Worker for private comp.	695 (46.27)	70.6	29.4			
Children						2.222 (1.873, 2.639)
No	2047 (63.43)	83.9	16.1	85.359	<0.001	
Yes	1180 (36.57)	70.1	29.9			
Pet						1.081 (0.898, 1.301)
Yes	2304 (71.40)	79.2	20.8	0.680	0.410	
No	923 (28.60)	77.9	22.1			
Disability						1.342 (0.844, 2.137)
No	3132 (97.06)	79.0	21.0	1.556	0.212	
Yes	95 (2.94)	73.7	26.3			
Health worker						1.385 (1.062, 1.580)
No	2543 (78.8)	79.8	20.2	6.528	0.011	
Yes	684 (21.2)	75.3	24.7			
Confinement						-
Strict	843 (26.1)	81.0	19.0	24.200	<0.001	
Except for purchase-work	1793 (55.6)	80.0	20.0			
No	326 (10.1)	69.0	31.0			
Other situations	265 (8.2)	75.8	24.2			

* Grouped variable from median value.

**Table 2:** Psychological Distress: General Health Questionnaire GHQ-12.

	TOTAL (*N* = 3227)
Item	M (SD)
1. Have you been able to concentrate well on what you were doing?	2.86 (0.80)
2. Have your worries made you lose a lot of sleep?	2.82 (0.99)
3. Have you felt that you are playing a useful role in life?	2.36 (0.96)
4. Have you felt capable of making decisions?	2.29 (0.80)
5. Have you felt constantly overwhelmed and stressed?	3.08 (0.91)
6. Have you had the feeling that you cannot overcome your difficulties?	2.52 (1.01)
7. Have you been able to enjoy your normal daily activities?	2.92 (0.89)
8. Have you been able to adequately cope with problems?	2.47 (0.79)
9. Have you felt unhappy or depressed?	2.73 (1.01)
10. Have you lost confidence in yourself?	2.15 (1.08)
11. Have you thought that you are a worthless person?	1.68 (1.01)
12. Do you feel reasonably happy considering all the circumstances?	2.37 (0.85)
GHQ-12 (over 12 points)	6.16 (3.76)
Cut-off point ≥ 3	N (%)
Yes	2544 (78.83)
No	683 (21.17)

α-Cronbach = 0.910 Items 1 to 12 rank 1–4.

**Table 3:** Association between self-perceived health, physical symptoms, and other health variables related with psychological distress during the COVID-19 pandemic.

	TOTAL (*N* = 3227)					
		GHQ				
	*N* (%)	YES (*N* = 2544)	NO (*N* = 683)	χ2	*p*	Odds Ratio (CI = 95%)
PHYSICAL SYMPTOMS						
Fever						2.221 (0.874, 5.643)
Yes	46 (1.4)	89.1	10.9	2.965	0.085	
No	3181 (98.6)	78.7	21.3			
Cough						1.715 (1.354, 2.172)
Yes	647 (20.0)	85.3	14.7	20.378	<0.001	
No	2580 (80.0)	77.2	22.8			
Headache						3.183 (2.660, 3.810)
Yes	1731 (53.6)	87.5	12.5	168.868	<0.001	
No	1496 (46.4)	68.8	31.2			
Muscle pain						2.662 (2.105, 3.366)
Yes	845 (26.2)	89.0	11.0	70.810	<0.001	
No	2382 (73.8)	75.2	24.8			
Dizziness						2.936 (2.031, 4.243)
Yes	363 (11.2)	90.9	9.1	35.737	<0.001	
No	2864 (88.8)	77.3	22.7			
Diarrhea						3.213 (2.214, 4.663)
Yes	379 (11.7)	91.6	8.4	41.655	<0.001	
No	2848 (88.3)	77.1	22.9			
Sore throat						1.866 (1.460, 2.385)
Yes	625 (19.4)	86.2	13.8	25.474	<0.001	
No	2602 (80.6)	77.1	22.9			
Coryza						1.949 (1.606, 2.366)
Yes	1122 (34.8)	85.6	14.4	46.644	<0.001	
No	2105 (65.2)	75.2	24.8			
Chills						2.144 (1.412, 3.256)
Yes	225 (7.0)	88.4	11.6	13.386	<0.001	
No	3002 (93.0)	78.1	21.9			
Breathing difficulty						2.154 (1.271, 3.650)
Yes	141 (4.4)	88.7	11.3	8.517	0.004	
No	3086 (95.6)	78.4	21.6			
CURRENT HEALTH STATUS						
Self-perceived health						6.803 (4.808, 9.524)
Fair/bad/very bad	749 (23.2)	95.1	4.9	153.894	<0.001	
Excellent/good/very good	2478 (76.8)	73.9	26.1			
Chronic diseases						1.067 (0.888, 1.283)
Yes	945 (29.3)	78.1	21.9	0.438	0.508	
No	2282 (70.7)	79.1	20.9			
Currently taking any medication						1.083 (0.911, 1.289)
No	2026 (62.8)	79.3	20.7	0.764	0.382	
Yes	1201 (37.2)	78.0	22.0			
Hospitalised in the last 14 days						1.333 (0.479, 3.717)
No	3208 (99.4)	78.9	21.1	0.304	0.581	
Yes	19 (0.6)	73.7	26.3			
Health care in the last 14 days						1.291 (0.937, 1.779)
Yes	281 (8.7)	82.6	17.4	2.563	0.109	
No	2946 (91.3)	78.5	21.5			

**Table 4:** Association between variables related with history of contact and psychological distress during the pandemic.

	TOTAL (*N* = 3227)					
		GHQ				
	*N* (%)	Yes (*N* = 2544)	No (*N* = 683)	Statistical	*p*	Odds Ratio (CI = 95%)
Contact >15′ <2 m with infected person						1.372 (1.105, 1.704)
Yes, or doesn’t know	708 (21.9)	82.8	17.2	8.411	0.004	
No	2519 (78.1)	77.7	22.3			
Casual contact with infected person						1.453 (1.190, 1.772)
Yes, or doesn’t know	907 (28.1)	83.0	17.0	13.250	<0.001	
No	2320 (71.9)	77.2	22.8			
Contact with any person or material suspicious of being infected						1.485 (1.240, 1.777)
Yes, or doesn’t know	1248 (38.7)	82.8	17.2	18.910	<0.001	
No	1979 (61.3)	76.4	23.6			
Any infected family member						1.320 (1.038, 1.679)
Yes, or doesn’t know	537 (16.6)	82.5	17.5	5.173	0.023	
No	2690 (83.4)	78.1	21.9			
Having been performed diagnostic test						1.153 (0.912, 1.460)
No	2767 (85.7)	79.2	20.8	1.412	0.235	
Yes	460 (14.3)	76.7	23.3			

**Table 5:** Contrast between preventive measures and psychological distress during the pandemic.

	TOTAL(*N* = 3227)				
		GHQ			
	M (SD)	Yes (*N* = 2544)	No (*N* = 683)	Statistical	*p*
Covering mouth	4.56 (0.76)	4.55 (0.76)	4.60 (0.75)	−1.411	0.158
Avoiding sharing utensils	4.29 (1.16)	4.25 (1.17)	4.43 (1.10)	−3.784	<0.001
Washing hands with soap and water	4.75 (0.52)	4.74 (0.53)	4.79 (0.47)	−2.250	0.025
Washing hands with hydroalcoholic solution	3.89 (1.12)	3.86 (1.12)	4.00 (1.12)	−2.994	0.003
Washing hands immediately after coughing, touching the nose, or sneezing	3.60 (1.18)	3.56 (1.17)	3.75 (1.18)	−3.675	<0.001
Washing hands after touching potentially contaminated objects	4.57 (0.75)	4.56 (0.76)	4.62 (0.72)	−1.710	0.087
Wearing a mask regardless of the presence of symptoms	4.77 (0.62)	4.77 (0.62)	4.77 (0.61)	−0.303	0.762
Leaving at least a metre and a half distance	4.51 (0.67)	4.49 (0.68)	4.58 (0.65)	−3.157	0.002

Note: Likert-type response scale from 1 (Never) to 5 (Always).

## Data Availability

All data are available within this article.
